# Accelerated amyloid angiopathy and related vascular alterations in a mixed murine model of Alzheimer´s disease and type two diabetes

**DOI:** 10.1186/s12987-022-00380-6

**Published:** 2022-11-07

**Authors:** Maria Vargas-Soria, Juan Jose Ramos-Rodriguez, Angel del Marco, Carmen Hierro-Bujalance, Maria Jose Carranza-Naval, Maria Calvo-Rodriguez, Susanne J. van Veluw, Alan W. Stitt, Rafael Simó, Brian J. Bacskai, Carmen Infante-Garcia, Monica Garcia-Alloza

**Affiliations:** 1 Division of Physiology. School of Medicine, University of Cadiz, Cadiz, Spain; 2 Instituto de Investigacion e Innovacion en Ciencias Biomedicas de la Provincia de Cadiz (INIBICA), Cadiz, Spain; 3 Currently at Department of Physiology, School of Health Sciences, University of Granada, Granada, Spain; 4 Salus-Infirmorum, University of Cadiz, Cadiz, Spain; 5 Alzheimer Research Unit, Department of Neurology, Massachusetts General Hospital and Harvard Medical School, Boston, USA; 6 Wellcome-Wolfson Institute for Experimental Medicine, Queen’s University Belfast, Belfast, Northern Ireland UK; 7 Diabetes and Metabolism Research Unit, Vall d’Hebron Research Institute, Universitat Autonoma de Barcelona, Barcelona, Spain; 8 Centro de Investigacion Biomedica en Red de Diabetes y Enfermedades Metabolicas Asociadas (CIBERDEM), Instituto de Salud Carlos III (ISCIII), Madrid, Spain

**Keywords:** Alzheimer’s disease, Type 2 diabetes, Prediabetes, Multiphoton microscopy, Amyloid, Oxidative stress, Matrix metalloproteinases

## Abstract

**Background:**

While aging is the main risk factor for Alzheimer´s disease (AD), emerging evidence suggests that metabolic alterations such as type 2 diabetes (T2D) are also major contributors. Indeed, several studies have described a close relationship between AD and T2D with clinical evidence showing that both diseases coexist. A hallmark pathological event in AD is amyloid-β (Aβ) deposition in the brain as either amyloid plaques or around leptomeningeal and cortical arterioles, thus constituting cerebral amyloid angiopathy (CAA). CAA is observed in 85–95% of autopsy cases with AD and it contributes to AD pathology by limiting perivascular drainage of Aβ.

**Methods:**

To further explore these alterations when AD and T2D coexist, we have used in vivo multiphoton microscopy to analyze over time the Aβ deposition in the form of plaques and CAA in a relevant model of AD (APPswe/PS1dE9) combined with T2D (db/db). We have simultaneously assessed the effects of high-fat diet-induced prediabetes in AD mice. Since both plaques and CAA are implicated in oxidative-stress mediated vascular damage in the brain, as well as in the activation of matrix metalloproteinases (MMP), we have also analyzed oxidative stress by Amplex Red oxidation, MMP activity by DQ^™^ Gelatin, and vascular functionality.

**Results:**

We found that prediabetes accelerates amyloid plaque and CAA deposition, suggesting that initial metabolic alterations may directly affect AD pathology. T2D significantly affects vascular pathology and CAA deposition, which is increased in AD-T2D mice, suggesting that T2D favors vascular accumulation of Aβ. Moreover, T2D synergistically contributes to increase CAA mediated oxidative stress and MMP activation, affecting red blood cell velocity.

**Conclusions:**

Our data support the cross-talk between metabolic disease and Aβ deposition that affects vascular integrity, ultimately contributing to AD pathology and related functional changes in the brain microvasculature.

## Background

Alzheimer´s disease (AD) is the most common cause of dementia. Classical neuropathological features include aberrant amyloid and tau deposition in the brain that leads to a progressive neuronal and synaptic loss [1]. Amyloid pathology involves amyloid-β (Aβ) peptide, derived from proteolytic processing of the transmembrane amyloid precursor protein. As a pathogenic hallmark of AD, Aβ can form amyloid plaques and/or deposit around leptomeningeal and cortical arterioles, as cerebral amyloid angiopathy (CAA) [2]. These vascular deposits are uncommon in other brain areas such as the hippocampus, cerebellum, basal ganglia, thalamus, brain stem or white matter [3, 4]. Both types of Aβ deposit can occur either relatively independently of each other or they can overlap [2]. Offering conclusive evidence, a recent meta-analysis has reported that when based on neuropathological examination, the prevalence of moderate to severe CAA in AD is 48%, and 23% in the general population [5]. CAA worsens AD pathology by limiting perivascular drainage, a major pathway for Aβ clearance out of the brain [2].

Previous work has supported involvement of parenchymal Aβ and CAA [6] in oxidative stress-mediated vascular damage in the brain [7]. This may ultimately contribute to CAA-related cerebrovascular dysfunction, cerebral hemorrhage, and further increase of CAA [6, 8]. Other underlying mechanisms include altered matrix metalloproteinases (MMP) activity in association with CAA. MMP2 and MMP9 are affected in AD mice that reproduce amyloid pathology, and have been related to tight junction disruption and increase in blood–brain barrier (BBB) permeability [9]. Also, Aβ oligomers have been directly related to pericyte contraction which led to capillary stenosis, thus resulting in a reduction of the cerebral blood flow [7]. Although age remains the main risk factor for AD, advances in dementia research support that metabolic disorders also play an important role in the development of AD. Insulin resistance, hyperinsulinemia and type 2 diabetes (T2D) are metabolic alterations strongly associated with AD [10, 11]. The prevalence of metabolic disorders has continued to increase over the last years. The latest edition of the Diabetes Atlas shows that 463 million adults are currently living with diabetes worldwide [12], and T2D accounts for the vast majority of the cases (90% approximately). Sedentary habits and progressive aging of the population contribute to the current diabetes pandemic that is usually preceded by a prediabetic state. Prediabetes includes impaired glucose tolerance and/or impaired fasting glucose that leads to maintained hyperinsulinemia and translates to an increased risk of the future development of T2D.

Prediabetes and T2D lead to several macro and microvascular complications resulting in cardiovascular and cerebrovascular diseases. Specifically in the brain, these conditions can cause neuronal pathology, alterations in synaptic communication as well as amyloid and tau pathologies [13, 14]. Moreover, diabetes and even prediabetes impair memory processing [15]. Whereas all these data support common links between AD and metabolic disorders, to our knowledge the impact of prediabetes and overt T2D on vascular function, oxidative stress or MMP alterations associated with amyloid pathology in AD remains largely unexplored. Therefore, we have analyzed amyloid deposition in vivo, using longitudinal multiphoton microscopy, in murine models that harbor AD and prediabetes or T2D.

## Methods

### Animals and treatments

Prediabetes was induced by administering high-fat diet (HFD) (60% Kcal from fat, OpenSource, New Brunswick, NJ, USA) to wildtype and APP/PS1 immediately after weaning (3 weeks of age) and up to the end of the experiments by 32 weeks of age. These become overweight and present hyperinsulinemia, indicative of the prediabetic estate [16]. Untreated animals received regular diet (RD) (SAFE A04. Augy, France). Imaging acquisition started at 26 weeks of age and amyloid deposition was followed up to 32 weeks of age. MMP and oxidative stress were analyzed also at 32 weeks of age.

AD-T2D mice (APP/PS1xdb/db) were produced by crossbreeding APPswe/PS1dE9 mice [17] with db/db mice [18] as described [19]. Plaques can be detected by 6 months of age in APP/PS1 mice, when exponential amyloid deposition commences [20]. Therefore, the direct effect of T2D on amyloid deposition in APP/PS1xdb/db mice was analyzed from a slightly earlier stage, at 22 weeks of age, and up to 32 weeks of age. Since different genetic backgrounds can significantly determine pathological outcomes, studies on prediabetes and T2D animals (on C57BL/6xC3H and C57BLKS/J backgrounds, respectively) were performed in parallel, unless initial comparison between wildtype mice could be performed (metabolic studies) and no differences were detected. All studies were approved by the Animal Care and Use Committee of the University of Cadiz in accordance with the guidelines for care and use of experimental animals (European Commission Directive 2010/63/UE and Spanish Royal Decree 53/2013).

### Metabolic assessment

Body weight, glucose and insulin levels were determined in at 30–32 weeks of age, before sacrifice [14]. Blood glucose levels were measured using the glucometer Optium Xceed (Abbott, UK) and plasma insulin samples were collected into tubes with potassium-EDTA (Sarstedt, Nümbrecht, Germany). Blood samples were centrifuged during 7 min, 6500 rpm at 4 °C, and plasma fraction was stored at −80 °C until processed. Plasma insulin levels were measured using ultrasensitive mouse enzyme-linked immunosorbent assay (ALPCO Diagnostics, Salem, NH, USA).

### Oligomeric Aβ, Aβ40 and Aβ42

Immediately after the completion of the last imaging session, animals received an overdose of pentobarbital (100 mg/kg). Brains were harvested, right hemispheres were fixed in PFA and left hemispheres were dissected. Cortex was fresh frozen at −80 ºC until used. Human Amyloid β Oligomers (82E1-specific) Assay Kit (IBL International Corp, Hamburg, Germany) was used for Aβ oligomers quantification in the cortex. Tissue was homogenized (1/5 w/v) in Tris-buffered saline (TBS; 20 mM Tris–HCl, 140 mM NaCl, pH 7.5) with Halt™ phosphatase and protease inhibitor cocktail (Thermo Fisher Scientific Slu., Madrid, Spain). Homogenates were centrifuged (14,000 rpm) for 60 min at 4 °C. The supernatant was diluted 1:2 with EIA buffer from the kit, following manufacturer´s indications. Aβ40 and Aβ42 levels were also quantified in the cortex by colorimetric ELISA kits (Wako, Japan) as previously described [21]. Tissue (5–10 mg) was homogenized in 50 μl of lysis buffer with Halt^™^ phosphatase and protease inhibitor cocktail and centrifuged (14,000 rpm) for 12 min at 4 °C. Supernatants were used to measure soluble Aβ40 and Aβ42. The pellet was extracted with 65 μl of 70% formic acid and centrifuged (14,000 rpm) for 10 min. Samples were then neutralized with 1 M Tris (pH 11) and diluted 1:40 to measure insoluble Aβ levels. Human Aβ40 and Aβ42 provided in the kit were used for standard curves. All absorbances were measured spectrophotometrically at 450 nm (MQX200R2, Biotek instruments, Burlington VT, USA) and data were expressed as pmol/g tissue.

### Lipid peroxidation and DNA oxidation

Cortical lipid peroxidation was assessed by malondialdehyde (MDA) content following manufacturer´s indications (Lipid Peroxidation Assay Kit, Abcam, Cambridge, UK). Absorbances were measured spectrophotometrically at 532 nm. Data were expressed in nmol/g tissue.

DNA was purified from 5–10 mg cortical tissue by QIAamp DNA Mini Kit (Qiagen, MD, USA) according to the manufacturer’s instructions. DNA oxidation was analyzed by 8-hydroxy-2'deoxyguanosine (8-OHdG) content following manufacturer´s indications (8-OHdG ELISA Kit, Molecular Signature^®^, BC, Canada) and measuring the absorbance at 450 nm. 500 ng DNA per sample were used and data were expressed in ng 8-OHdG/mg DNA.

### Cranial window implantation and multiphoton imaging

Animals had permanent cranial windows placed for chronic in vivo imaging at 26 weeks of age in AD-HFD and 22 weeks of age in case of AD-T2D studies, as previously described [20]. To assess amyloid plaques and CAA deposition mice were imaged immediately after the surgery and allowed to recover. They were also reimaged every 2 weeks up to 32 weeks of age, or until regrowth of the underlying tissue did not allow the imaging session. All animals were imaged at least twice and isoflurane anesthesia was used in all imaging sessions. Aβ deposition was visualized with methoxy-XO4 (Abcam, Cambridge, UK) intraperitoneally injected (5 mg/Kg) the day before each imaging session. Angiograms were performed with 80 µl (12.5 mg/ml) i.v. retro-orbital injection of Texas Red dextran 70KD (Thermo Fischer Scientific, Spain) preceding the imaging session. In vivo imaging was conducted using a 20X (1.05 NA, Olympus) water immersion objective in a Fluoview 1000 MPE microscope (Olympus, Spain). Two-photon fluorescence was generated with 800 nm excitation, and collected emitted light in the range of 380–480, 500–540 and 560–650 nm. Along with the last imaging session, at 32 weeks of age, red blood cell (RBC) velocity was assessed in 29–94 segments per group, using repeated line scans of the same area at 800 nm (5X zoom) through the horizontal part of the selected region of interest as previously described [22] and measured [23] using ImageJ software. Vessel diameter changes [24] was also analyzed at 32 weeks of age, with 3 s line scans, and data were expressed as % diameter change for each vessel. For Aβ deposition 615 × 615 μm fields, z/step 5 μm, depth ~ 200 μm were imaged. Maximum intensity projections of z-series were obtained using ImageJ software. All stacks had 40 sections and were used to measure plaque size and number after 2D projections. Plaque size was measured by thresholding, segmenting and measuring using the blue fluorescence generated by methoxy-XO4. CAA burden was also analyzed in maximum intensity projections. Vessels were outlined and the CAA deposits were manually threshold using Image J free software, as previously described [6, 25]. The number of pixels in white clusters composed of at least 6 contiguous pixels was counted. The CAA burden was calculated as the percentage of the vessel area affected by CAA. New growth of CAA deposits was measured every 2 weeks, using the same approach. Texas-red signal was used to measure the vessel area from the initial imaging session as described [25, 26]. Vessel diameter was measured at 3 separate points along the vessel segment, perpendicular to the axis. We cannot obviate the effect that metabolic disorders may have in the surgeries, nevertheless the percentage of animals that underwent the complete procedure for the long-term in vivo imaging studies was similar in all groups under study (APP/PS1-RD 75%, APP/PS1-HFD 75%, APP/PS1 71% and APP/PS1xdb/db 75%).

A second set of APP/PS1-HFD and APP/PS1xdb/db mice were imaged to analyze other functional alterations associated with Aβ deposition and vascular damage. Oxidative stress and MMP activity were measured using Amplex Red (10-acetyl-3,7-dihydroxyphenoxazine) (Thermofisher, Spain), and the green fluorescent substrate DQ^™^ Gelatin (Thermofisher, Spain) respectively, while thioflavin S (Sigma, St. Louis, MO) was also used for histochemical confirmation. The fluorophores were imaged in three immediately consecutive sessions so the signal detected was exclusively derived from the marker under study at each time, as previously described [6, 27]. Briefly, 32 weeks old animals were anesthetized as described above, the dura was removed and 10 μl of Amplex Red in 85 μl of filtered PBS (1 mM) with 0.5 mg/ml peroxidase was locally applied for 20 min. A coverslip (8 mm in diameter) was placed, and animals were imaged. The coverslip was removed and 50 μl of the green fluorescent substrate DQ^™^ Gelatin (1 mg/ml) was applied for 20 min. After the second imaging session animals were incubated with thioflavin S (0.1 mg/ml) for 10 min before the final imaging session. Amyloid plaques and CAA imaging was performed as described above and images were analyzed using Image J [6, 27]. Vessels were outlined and Amplex Red, DQ^™^ Gelatin and thioflavin S signals (oxidative stress, MMP activity and Aβ deposits respectively) were quantified. In each vessel segment, Amplex Red, the green fluorescent substrate DQ^™^ Gelatin and thioflavin S signal were threshold and signal-positive regions were quantified. The number of continuous pixels larger than 6 were counted. In each vessel segment, Amplex Red and DQ^™^ Gelatin signals were expressed as a percentage of thioflavin S signal [6]. Image volumes focused on leptomeningeal vessels and not parenchymal plaques, in order to obtain sufficient high quality images, however, a small set of amyloid plaques were analyzed and Amplex Red and MMP intensities were measured and normalized to thioflavin S intensity [27].

### Statistical analysis

One-way ANOVA for independent samples followed by Tukey B test or Tamhane tests was used when four groups were under study (metabolic assessment, RBC velocity and vessel diameters), and Student t test for independent samples was used when only two groups were compared (Aβ levels, plaques, CAA, oxidative stress and MMP activation). Two-way ANOVA (groupXweek) was used to analyze amyloid plaques and CAA progression rates. SPSS v.24 software was used for all statistical analysis and results are expressed as mean ± SEM.

## Results

### Metabolism

Initial comparison between wildtype mice from AD-T2D and AD-HFD studies revealed no differences when body weight (p = 0.231), glucose levels (p = 0.187) or insulin levels (p = 0.746) were compared. Hence, these mice were pooled and considered as the Control group of the study in the following analyses. Body weight was significantly increased in APP/PS1 mice and their wildtype littermates on HFD, db/db, and APP/PS1xdb/db mice compared to Control mice [14, 19] (Table 1). Glucose levels were significantly higher in diabetic mice (db/db and APP/PS1xdb/db) when compared with Control and APP/PS1 animals, while insulin levels were significantly increased in diabetic (db/db and APP/PS1xdb/db mice) and prediabetic (Control-HFD and APP/PS1-HFD mice (Table 1).

**Table 1:** Metabolic assessment

	Body weight (g)	Glucose (mg/dl)	Insulin (ng/dl)
Control	28.50 ± 1.84	122.71 ± 9.58	0.75 ± 0.29
Control-HFD	44.50 ± 4.80††	114.00 ± 7.15	5.52 ± 2.28††
APP/PS1	28.61 ± 0.87	129.83 ± 4.20	0.69 ± 0.12
APP/PS1-HFD	42.95 ± 2.54††	118.17 ± 9.94	7.13 ± 0.99††
db/db	48.88 ± 2.83††	484.50 ± 33.73‡‡	6.90 ± 1.76††
APP/PS1xdb/db	42.87 ± 2.09††	448.40 ± 68.20‡‡	6.68 ± 2.08††

Body weight [F_(5,35)_ = 13.04, ††p < 0.01 vs. Control and APP/PS1] and insulin levels [F_(5,44)_ = 5.32, ††p < 0.01 vs. Control and APP/PS1] were significantly increased in animals on HFD, db/db and APP/PS1xdb/db mice, while glucose levels were significantly higher in diabetic mice [F_(5,39)_ = 52.19, ‡‡p < 0.01 vs. Control, Control-HFD, APP/PS1 and APP/PS1-HFD]. Data are representative of 5–12 mice (Control n = 7, Control-HFD n = 7, APP/PS1 n = 12, APP/PS1-HFD n = 6, db/db n = 8, APP/PS1xdb/db n = 5)

### Aβ40, Aβ42 and oligomeric Aβ

**Fig. 1:**
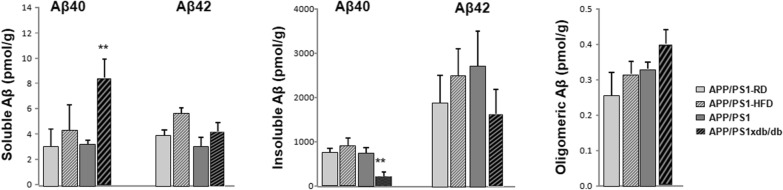
Aβ levels are altered in prediabetic and T2D mice. Soluble Aβ40 is increased in APP/PS1xdb/db mice when compared with APP/PS1 and prediabetic-APP/PS1 animals, [F_(3,44)_ = 5.03, **p = 0.004 vs. rest of the groups] while differences did not reach statistical significance for soluble Aβ42 [F_(3,36)_ = 2.20, p = 0.105]. On the other hand insoluble Aβ40 is reduced in APP/PS1xdb/db mice [F_(3,40)_ = 5.064 **p = 0.003 vs. rest of the groups] and a similar trend is observed for insoluble Aβ42 although differences are not statistically significant [F_(3,27)_ = 0.798, p = 0.506] (APP/PS1-RD n = 9–12, APP/PS1-HFD n = 7–12, APP/PS1 n = 8–12 and APP/PS1xdb/db n = 7–12) (mean ± SEM). While oligomeric Aβ is slightly higher in APP/PS1xdb/db mice, differences are not statistically significant [F_(3,16)_ = 1.25, p = 0.323] (APP/PS1-RD n = 5, APP/PS1-HFD n = 5, APP/PS1 n = 4 and APP/PS1xdb/db n = 6) (mean ± SEM)

A slight increase in Aβ40 and Aβ42 levels was observed in the cortex from APP/PS1 mice on HFD when compared with animals treated with RD, although differences were not statistically significant (Fig. 1). As previously [14, 19], we observed a shift in Aβ levels; soluble Aβ was increased in APP/PS1xdb/db mice, reaching statistic significance in case of Aβ40 levels, while insoluble levels were reduced, especially when insoluble Aβ40 was assessed. No differences were detected when oligomeric Aβ levels were measured (Fig. 1).

### CAA deposition is increased in larger caliber vessels from AD- T2D mice

**Fig. 2:**
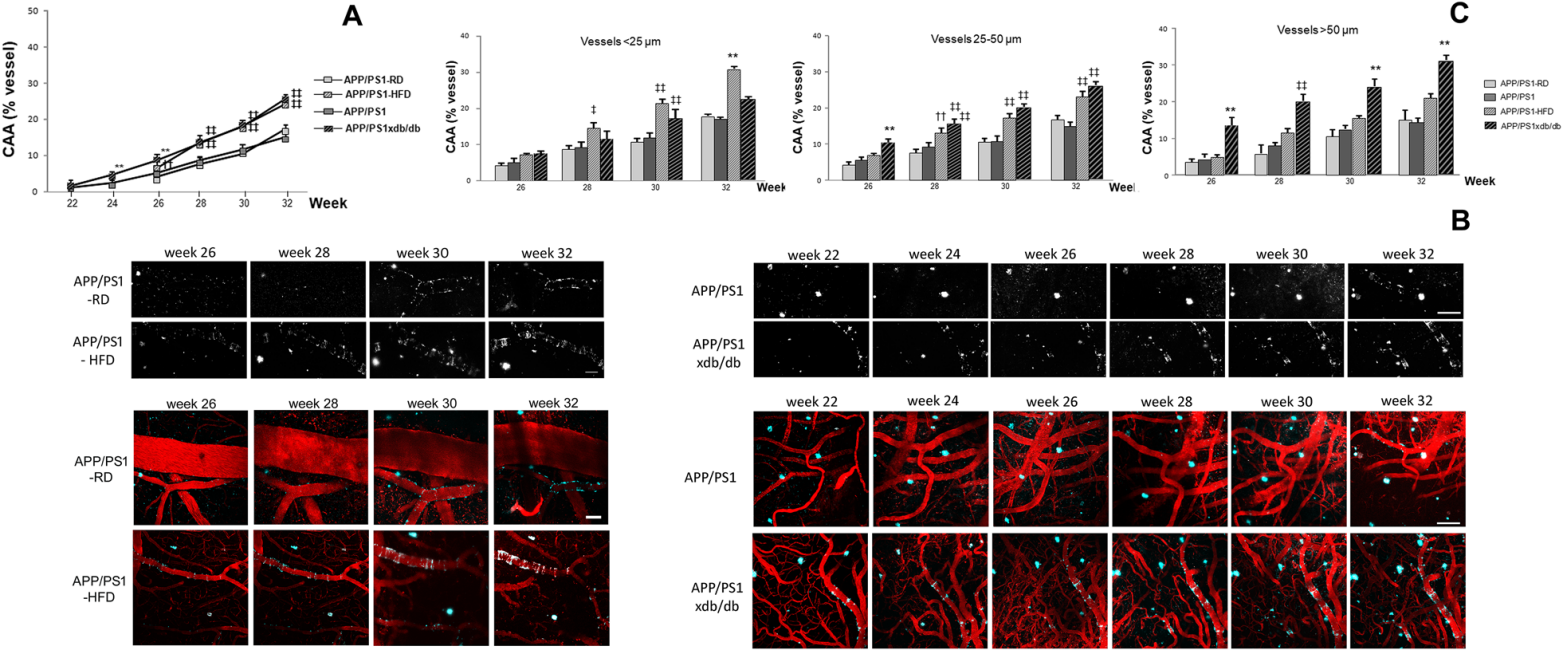
Prediabetes and T2D accelerate CAA progression rates in APP/PS1 mice in vivo and in real time. **A** Assessment of CAA deposition revealed that HFD-induced prediabetes as well as T2D significantly increased CAA deposition as disease progressed. When individual imaging sessions were compared no differences were detected between APP/PS1 and APP/PS1xdb/db mice at 22 weeks of age (p = 0.099), however significant differences were observed at 24 weeks of age when APP/PS1 and APP/PS1xdb/db mice were compared (**p < 0.01 vs. APP/PS1). By 26 weeks of age CAA burden was higher in APP/PS1-HFD animals and even higher burdens were observed in APP/PS1xdb/db mice [F_(3,690)_ = 15.056, **p < 0.001 vs. rest of the groups; ††p < 0.01 vs. APP/PS-RD]. From 28 weeks of age on, both HFD-induced prediabetes and T2D significantly increased CAA deposition in APP/PS1 animals (week 28 [F_(3,617)_ = 18.42, ‡‡p < 0.0.1 vs. APP/PS1-RD and APP/PS1]; week 30 [F_(3,589)_ = 25.98, ‡‡p < 0.0.1 vs. APP/PS1-RD and APP/PS1]; week 32 [F_(3,503)_ = 26.16, ‡‡p < 0.0.1 vs. APP/PS1-RD and APP/PS1]) (APP/PS1-RD n = 4 and 42–81 vessel segments, APP/PS1-HFD n = 4 and 86–118 vessels segments, APP/PS1-RD n = 7 and 160–262 vessel segments, APP/PS1xdb/db and 233vessel segments) (mean ± SEM). **B** Illustrative example of individual vessels labeled with methoxy-XO4 to quantify CAA deposition along imaging sessions. CAA is isolated from the background and set white in binary images. Scale bar = 100 µm. Illustrative pseudocolor images of cortical regions (upper panel) were used to analyze CAA deposition along the biweekly imaging sessions in all groups under study (bottom panel). Blood vessels are labeled in red (Texas Red dextran 70KD) and amyloid in blue (methoxy-XO4). Scale bar = 100 µm. **C)** CAA deposition was increased in smaller vessels from APP/PS1-HFD mice whereas larger vessels from AD-T2D mice were more severely affected (diameter ≤ 25 µm: 26 weeks of age [F_(3,256)_ = 2.49, p = 0.061], 28 weeks [F_(3,224)_ = 3.89, ‡p = 0.010 vs. APP/PS1-RD and APP/PS1], 30 weeks [F_(3,256)_ = 9.67, ‡‡p < 0.01 vs. APP/PS1-RD and APP/PS1], 32 weeks [F_(3,250)_ = 10.71, **p < 0.01 vs. rest of the groups]; diameter  > 25 ≤ 50 µm: 26 weeks of age [F_(3,267)_ = 7.86, **p < 0.01 vs. rest of the groups], 28 weeks [F_(3,236)_ = 8.13, ‡‡p < 0.01 APP/PS1-RD and APP/PS1, ††p < 0.01 vs. APP/PS1-RD], 30 weeks [F_(3,223)_ = 13.94, ‡‡p < 0.01 APP/PS1-RD and APP/PS1], 32 weeks [F_(3,174)_ = 18.44, ‡‡p < 0.01 APP/PS1-RD and APP/PS1]; diameter > 50 µm: 26 weeks of age [F_(3,159)_ = 14.39, **p < 0.01 vs. rest of the groups], 28 weeks [F_(3,149)_ = 14.82, ‡‡p < 0.01 vs. APP/PS1-RD and APP/PS1], 30 weeks [F_(3,102)_ = 8.78, **p < 0.01 vs. rest of the groups], 32 weeks [F_(3,71)_ = 12.79, **p < 0.01 vs. rest of the groups]) (mean ± SEM)

Assessment of CAA deposition revealed that HFD-induced prediabetes as well as T2D significantly increased CAA deposition as disease progressed and we detected a significant treatmentXweek effect [F_(11,3393)_ = 8.79, **p < 0.001] (statistical power 1.0) (Fig. 2A and B). Individual weekly assessment revealed increased CAA deposition in APP/PS1xdb/db mice by 24 weeks of age and this increase was observed up to 32 weeks of age. Also, long-term HFD significantly increased CAA deposition as disease progressed (Fig. 2A and B). Interestingly, we observed that while the prediabetic process seems to affect smaller caliber vessels (diameter  ≤ 25 µm) the diabetic process significantly increased CAA deposition in larger vessels. In vessels  > 25 and  ≤ 50 µm, the amount of CAA was increased in APP/PS1-HFD and more robustly in APP/PS1xdb/db mice and this effect was more severe in larger vessels from APP/PS1xdb/db mice (diameter  > 50 µm) (Fig. 2C).

### In vivo amyloid plaque deposition

**Fig. 3:**
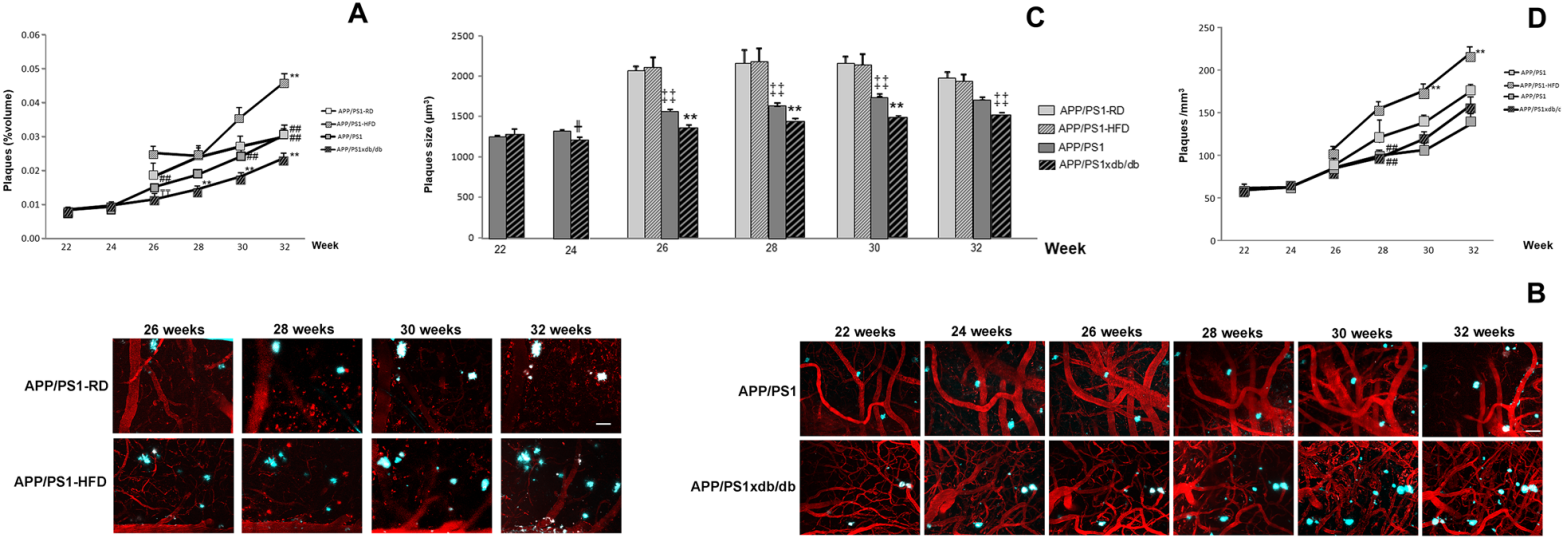
Long term HFD increases amyloid plaques deposition and T2D reduces amyloid plaques deposition in APP/PS1 mice. **A** Long-term HFD administration increased plaque burden (% of cortical volume) while it was significantly reduced in APP/PS1xdb/db mice. No differences were observed at 22 (p = 0.277) and 24 (p = 0.430) weeks of age. However, by 26 weeks of age amyloid plaque burden was significantly increased on animals on HFD and reduced burdens were observed in APP/PS1xdb/db animals [F_(3,311)_ = 13.447, ₸₸p < 0.001 vs. APP/PS1-RD and APP/PS1-HFD, ##p < 0.01 vs. APP/PS1-HFD] and similar profiles were observed afterwards (week 28 [F_(3,280)_ = 6.73, **p < 0.001 vs. rest of the groups], week 30 [F_(3,289)_ = 13.25, **p < 0.001 vs. rest of the groups, ##p < 0.01 vs. APP/PS1-HFD], week 32 [F_(3,299)_ = 17.99, **p < 0.001 vs. rest of the groups, ##p < 0.01 vs. APP/PS1-HFD]. **B** Illustrative images of cortical regions used to analyze plaque deposition in 32 week-old mice. Blood vessels are labeled in red (Texas Red dextran 70KD) and amyloid in blue (methoxy-XO4). Scale bar = 50 µm. **C** Amyloid plaques size was smaller in the mixed colony and individual biweekly assessment revealed that plaque size was even further reduced in APP/PS1xdb/db mice when compared with the rest of the groups (week 22 p = 0.561 vs. APP/PS1, week 24 ╫p = 0.021 vs. APP/PS1, week 26 [F_(3,1638)_ = 16.079, **p < 0.001 vs. rest of the groups, ‡‡p < 0.001 vs. APP/PS1-RD and APP/PS1-HFD], week 28 [F_(3,1638)_ = 15.371, **p < 0.001 vs. rest of the groups, ‡‡p < 0.001 vs. APP/PS1-RD and APP/PS1-HFD], week 30 [F_(3,1995)_ = 11.003, **p < 0.001 vs. rest of the groups, ‡‡p < 0.001 vs. APP/PS1-RD and APP/PS1-HFD], week 32 [F_(3,2980)_ = 5.076, ‡‡p < 0.001 vs. APP/PS1-RD and APP/PS1-HFD]). **D** Biweekly assessment revealed an increase in the number of new amyloid plaques in APP/PS1 animals on HFD when compared with the rest of the groups in the long term (week 22 p = 0.501, week 24 p = 0.976, week 26 [F_(3,315)_ = 2.35, p = 0.079], week 28 [F_(3,284)_ = 4.20, ##p = 0.006 vs. APP/PS1-HFD], week 30 [F_(3,302)_ = 8.76, **p < 0.001 vs. rest of the groups], week 32 [F_(3,316)_ = 27.70, **p < 0.001 vs. rest of the groups]) (APP/PS1-RD n = 4 and 47–65 fields, APP/PS1-HFD n = 4 and 49–79 fields, APP/PS1 n = 7 and 77–112 fields, APP/PS1xdb/db n = 4 and 47–91 fields) (mean ± SEM)

When we analyzed plaque burden (% of cortical volume) we observed that progression rates were lower in AD-T2D mice when compared with AD animals, in line with previous *postmortem* observations in this mouse model [14, 19], whereas plaque deposition rates were higher in prediabetes animals as previously observed in *postmortem* studies [16]. We detected a significant weekXgroup effect [F_(11,1465)_ = 2.43, p = 0.005] (statistical power 0.962) and individual biweekly assessment confirmed an accelerated increase in the rate of deposition after long-term HFD and lower deposition in APP/PS1xdb/db mice as disease progressed (Fig. 3A and B). We did not detect significant differences in amyloid plaque burden between APP/PS1 and APP/PS1-RD animals when all four groups under study were analyzed; nevertheless individual comparisons between these 2 groups revealed increased burdens in APP/PS1-RD mice at the beginning of the study (week 26 p = 0.021 and week 28 p = 0.016) when differences in amyloid plaque size are larger, however differences in amyloid plaque burden were not detected by 32 weeks of age, when amyloid plaque size is also similar in APP/PS1 and APP/PS1-RD.

In vivo multiphoton microscopy studies also allowed us to follow amyloid plaque size evolution. Plaque size followed a similar trend in all groups under study and, since plaque size remained constant along the experiments no significant groupXweek effect was observed [F_(11,9132)_ = 1.002, p = 0.441] (statistical power 0.573) in line with previous observations showing similar outcomes in APP/PS1 animals [28, 29]. However, as previously described amyloid plaques size was smaller in the mixed colony and individual biweekly assessment revealed that plaque size was even further reduced in APP/PS1xdb/db mice when compared with APP/PS1 mice (Fig. 3C). Whereas we cannot point towards a specific reason, it is feasible that using two mice colonies might account for the differences observed in amyloid plaque size and therefore control APP/PS1 mice are used for both colonies. Moreover, differences are observed when APP/PS1xdb/db and APP/PS1-HFD mice are compared with their respective controls.

In prediabetic mice, we observed that increased plaque burden was due to an increased deposition of new plaques and we detected a significant groupXweek effect [F_(11,1497)_ = 4.738, p < 0.001] (statistical power 1.000) when we analyzed amyloid plaque density. Biweekly assessment showed a maintained increase in the number of amyloid plaques in APP/PS1 animals on HFD, while no differences were observed when APP/PS1xdb/db mice were analyzed (Fig. 3D). These observations support that in APP/PS1xdb/db mice the reduction of plaque burden is due to the presence of smaller plaques, while the number of individual deposits is not affected, in line with previous studies showing lower plaque burden in this animal model [14].

### Vessel diameter and RBC velocity

**Fig. 4:**
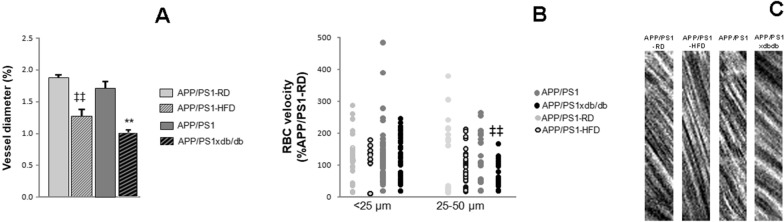
Vessel diameter change and RBC velocity are affected in diabetic APP/PS1 mice. **A** When we compared vessels with CAA, we observed a significant reduction in vessel diameter change in prediabetic APP/PS1 animals and larger reductions were observed in APP/PS1xdb/db mice [F_(3,1172)_ = 29.87, **p < 0.01 vs. rest of the groups, ‡‡p < 0.0.1 vs. APP/PS1-RD and APP/PS1]. Data are representative of 4–6 mice (APP/PS1 n = 6, APP/PS1xdb/db n = 4, APPP/PS1-RD n = 5, APP/PS1-HFD n = 5) (mean ± SEM). **B** A reduction of RBC velocity was observed in APP/PS1xdb/db mice that reached statistical significance when 25–50 µm in diameter vessels were analyzed. Whereas a similar profile was observed in prediabetic mice, differences did not reach statistical significance (< 25 µm [F_(3,160)_ = 0.589, p = 0.623]; 25–50 µm [F_(3,123)_ = 4.227, ‡‡p = 0.007 vs. APP/PS1-RD and APP/PS1]). Individual RBC are represented from 5–15 line scans (APP/PS1 < 25 µm n = 15, 25–50 µm n = 11, APP/PS1xdb/db < 25 µm n = 15, 25–50 µm n = 8, APP/PS1-RD < 25 µm n = 9, 25–50 µm n = 6, APP/PS1-HFD < 25 µm n = 5, 25–50 µm n = 9). **C**) Representative examples of RBC line scans in CAA vessels from APP/PS1-RD, APP/PS1-HFD, APP/PS1 and APP/PS1xdb/db mice

Besides structural alterations to the vasculature, CAA and diabetes also affect vessel function. To differentiate the role of diabetes and CAA on vessel function, we therefore measured the % vessel diameter change (generated by the heartbeat) in vessels with CAA in mice with and without prediabetes or diabetes under isoflurane anesthesia. A general decrease in vessel diameter change was observed in vessels affected by CAA in prediabetic AD animals. We observed larger reductions in vessel diameter change in APP/PS1xdb/db mice compared to AD mice (Fig. 4A). RBC velocity was reduced in small caliber vessels (25–50 µm) in APP/PS1xdb/db (i.e. AD-T2D mice) compared to APP/PS1 mice, but not in prediabetic mice (Figs. 4B and C). The number of vessels available for analysis precluded in depth assessment of RBC velocity in larger caliber vessels, although we observed no significant differences when APP/PS1 and APP/PS1-RD mice were compared (p = 0.121).

### Oxidative stress and MMP activation associated with amyloid deposition

**Fig. 5:**
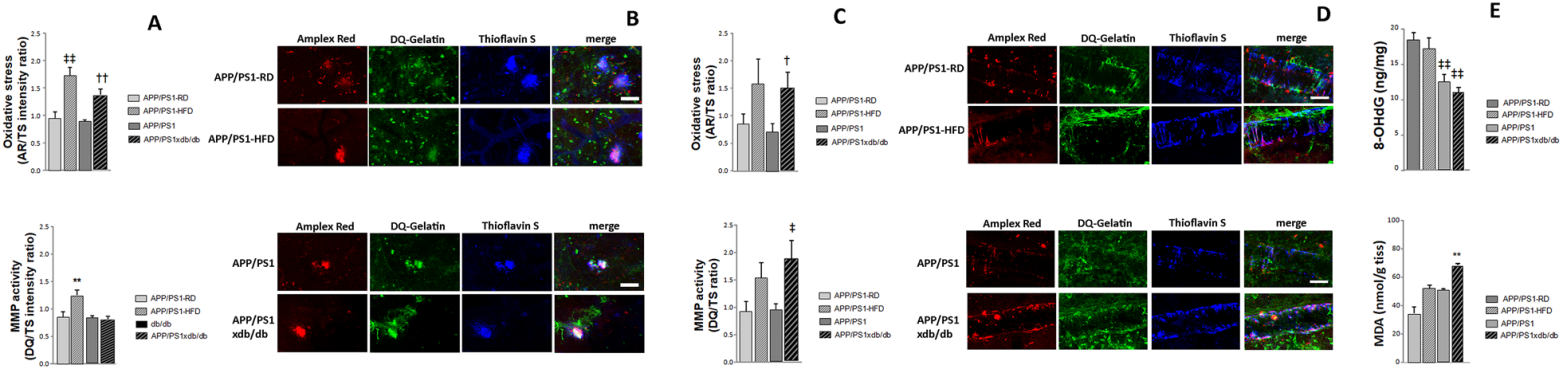
Oxidative stress and MMP activation are altered in prediabetic and diabetic APP/PS1 mice. **A** Oxidative stress associated with amyloid plaques (Amplex Red intensity/thioflavin intensity) was significantly increased in APP/PS1-HFD and APP/PS1xdb/db mice [F_(3,117)_ = 10.009, ‡‡p < 0.001 vs. APP/PS1-RD and APP/PS1, ††p < 0.01 vs. APP/PS1]. MMP activity was also significantly increased in APP/PS1-HFD mice in association with amyloid plaques [F_(3,126)_ = 7.71, **p < 0.001 vs. rest of the groups] (APP/PS1-RD n = 3 and 15–16 plaques, APP/PS1-HFD n = 4 and 20–22 plaques, APP/PS1 n = 7 and 50–56 plaques, APP/PS1xdb/db n = 6 and 40–41 plaques) (mean ± SEM). **B** Illustrative example of oxidative stress and MMP activation associated with amyloid plaques in all groups under study. Scale bar = 50 µm. **C** No differences were observed when oxidative stress or MMP activity were analyzed in association with CAA in prediabetic mice, although oxidative stress was significantly higher in APP/PS1xdb/db mice when compared with APP/PS1 animals [F_(3,387)_ = 3.32, †p = 0.019 vs. APP/PS1] and a significant increase in MMP activity was also observed in association with CAA in APP/PS1xdb/db mice [F_(3,283)_ = 3.88, ‡p = 0.01 vs. APP/PS1 and APP/PS1-RD] (APP/PS1-RD n = 3 and 69–72 vessel segments, APP/PS1-HFD n = 4 and 56–62 vessel segments, APP/PS1 n = 7 and 71–73 vessel segments, APP/PS1xdb/db n = 6 and 88–93 vessel segments) (mean ± SEM). **D** Illustrative example of oxidative stress and MMP activation associated with CAA in all groups under study. Scale bar = 50 µm. **E** 8-OHdG was increased in AD colony used to study the effect of prediabetes [F_(3,16)_ = 7.93, ‡‡p < 0.01 vs. APP/PS1-RD and APP/PS1-HFD] **F** Lipid peroxidation, estimated by MDA content, was significantly increased in APP/PS1xdb/db mice animals [F_(3,18)_ = 8.92, **p < 0.01 vs. rest of the groups]

Oxidative stress analyzed by Amplex Red intensity was measured in association with amyloid plaques and CAA in a separate cohort of mice at 32 weeks of age. Amplex Red intensity ratio (Amplex Red intensity/thioflavin S intensity) associated with amyloid plaques was significantly higher in APP/PS1xdb/db and APP/PS1-HFD (Fig. 5A and B). We also detected an increase in MMP activity, measured by DQ^™^ Gelatin signal, associated with plaques in HFD-treated mice (Fig. 5A and B). When we analyzed oxidative stress associated with CAA a significant increase was observed in APP/PS1xdb/db mice (Fig. 5C and D) and APP/PS1xdb/db mice also showed an increase in MMP intensity associated with CAA (Fig. 5C and D).

### Lipid peroxidation, DNA and protein oxidation

Next, we analyzed *postmortem* lipid and DNA oxidation by MDA and 8-OHdG content. 8-OHdG levels were higher in the AD colony used to study the effect of prediabetes. We cannot exclude that different colonies might account for detected differences, although we cannot point towards a specific cause for this dissimilarity. The fact that no differences were observed when APP/PS1-RD and APP/PS1-HFD mice were compared, or when APP/PS1xdb/db animals were compared with APP/PS1 animals, suggests a limited effect of metabolic alterations at this level (Fig. 5E). MDA content was significantly increased in the cortex from APP/PS1xdb/db mice when compared with APP/PS1 animals and APP/PS1-HFD animals (Fig. 5F).

## Discussion

Previous studies have reported the close relationship between AD and T2D, showing that the prevalence of mild cognitive impairment is high in T2D patients [30] and T2D might be a risk factor for AD. Alterations at the vascular level are a major link between AD and T2D [31] and it has been shown that the increased risk of clinically diagnosed AD might be mediated through cerebrovascular pathology [32]. Other potential links include insulin resistance due to insulin signaling impairment [10, 11, 33], which is also observed in prediabetes, inflammation or oxidative stress alterations [33]. These links may ultimately affect neurofibrillary tangles and amyloid deposition in AD and animal models that harbor AD and metabolic disorders, both prediabetes and overt T2D, have been developed [13, 14, 16, 34].

In line with previous studies in APP/PS1xdb/db mice [14, 19], we have observed a change in Aβ species, and soluble Aβ is increased while insoluble Aβ is reduced in AD-T2D mice. Other studies in AD models with metabolic disorders have also shown changes in amyloid deposition that do not affect total Aβ levels [13, 34] or amyloid burden. By contrast, studies with AD mice on HFD have shown increased levels of Aβ [16, 35], suggesting that prediabetes and overt T2D might not necessarily induce the same phenotypic changes. Since the actual changes in the natural history of Aβ deposition associated with metabolic alterations observed in APP/PS1xdb/db mice remain elusive, multiphoton microscopy could permit us to monitoring in vivo the deposition of amyloid plaques and CAA over time. Most AD patients present some grade of CAA, and direct vascular damage associated with metabolic disorders may contribute to alterations in Aβ deposition. Aβ40 is the main amyloid fragment present in vessel walls [36] and present understanding of CAA, although still controversial, has pointed out that Aβ from amyloid plaques in the brains of AD patients [37] may act as a "seed" and initiate the formation of vascular deposits of Aβ. Aβ40 is commonly found in the walls of leptomeningeal arteries and penetrating arterioles [2] and in the current study soluble Aβ40 levels were significantly increased in the cortex from AD-T2D mice in agreement with previous studies [34]. In vivo assessment of CAA deposition is significantly increased in APP/PS1 animals with T2D, when compared with APP/PS1 mice. Interestingly, the prediabetic process itself is enough to induce similar changes in CAA deposition. Previous *postmortem* studies have shown that CAA is increased in mixed models of AD and T2D [13] and vascular damage has also been observed in prediabetes mice [16]. While studies directly relating insulin or insulin resistance to CAA are scarce, our data suggest that altered insulin homeostasis could be a mediator of the observed vascular alterations. In this regard, memantine reduces CAA by enhancing insulin-degrading enzyme expression [38], supporting the deleterious cross-talk between insulin homeostasis and CAA. The fact that both prediabetic and AD-T2D mice show an increase in spontaneous cortical hemorrhages might also underlie both BBB dysfunction and impairment in Aβ drainage, as major mechanisms accounting for CAA [39].

When CAA-affected vessels were plotted by diameter size, in prediabetic mice we observed a preferential Aβ deposition in smaller vessels (< 25 µm) whereas Aβ deposition was significantly increased in larger vessels from AD-T2D mice. In line with these observations, Aβ is deposited in the walls of arteries and arterioles, and less frequently in capillaries and veins [40]. Also, studies focusing on other brain regions have reported that CAA preferentially affects larger vessels in the forebrain from transgenic mice, whereas smaller vessels are affected much later [41]. Differences in Aβ deposition at the vascular level have been previously described, and while Aβ40 tends to accumulate in leptomeningeal arteries and penetrating arterioles, capillaries tend to accumulate Aβ42, resembling the composition in plaques [2, 39]. The fact that Aβ42 is increased in our AD-HFD mice might support the detected differences with AD-T2D animals. Likewise, other studies have reported differential effects in vessels depending on APOE alleles. APOE ε4 inhibits Aβ transport across the BBB, it is associated with lower antioxidant activity and mediates BBB degradation through a proinflammatory pathway involving cyclophilin A in pericytes, impairing Aβ clearance at different levels [42]. Also, APOE ε4 has been related to CAA in capillaries and larger vessels, while APOE ε2 seems to affect larger arteriolar vessels whereas cortical capillaries are spared [39]. Nevertheless, further studies are needed to understand the link between APOE and T2D.

We also observed that while amyloid plaque numbers were increased in prediabetes animals, plaque size was not significantly affected, as previously observed in APP/PS1 animals [28] and this prediabetes model [16]. Aβ42 is more commonly observed in the parenchymal plaques of AD patients [39], and the fact that APP/PS1xdb/db mice have lower levels of Aβ42 than APP/PS1 animals, may account for this difference. Nevertheless, the actual composition of the plaques might not be static and amyloid plaque pathology in AD patients is generally heterogeneous when the morphologic characteristics of Aβ deposits are analyzed [43]. Interestingly, previous studies have shown that whereas T2D is a risk factor for AD and vascular dementia, the actual cerebral burden of the prototypical AD pathologies is not [44]. In line with this, our previous results also show similar outcomes when *postmortem* plaque burden is assessed [14, 19].

Vascular functionality was also examined in our animal models given the relevance of vascular risk factors in AD and their role in prediabetes and T2D. In this sense, vascular alterations have been proposed as feasible links contributing to an AD phenotype in diabetes, including vascular endothelial dysfunction, oxidative stress, inflammation, BBB dysfunction, neurovascular damage and MMP alterations [31, 44, 45]. In *postmortem* AD brains, tight junction proteins, such as occludin, claudin-5, and zonula occludens-1 are reduced in Aβ-loaded capillaries, and affected capillaries are associated with NOX-2-activated microglia and NADPH oxidase-2 [46]. Thus, neuroinflammation and reactive oxygen species contribute to Aβ endothelial toxicity and BBB disruption [46]. In capillaries isolated from AD mouse brains, degradation of P-glycoprotein and low-density lipoprotein receptor-related protein 1 is facilitated by Aβ40 exposure, which reduces the transport activity of P-glycoprotein and in turn the elimination of Aβ [47]. Also, in diabetic conditions, there is decreased expression of zonula occludens-1 in brain capillaries as well as increased perivascular immunoglobulin G leakage, indicating increased BBB permeability in diabetes [48].

Vessel diameter changes were reduced in CAA-affected vessels from AD-HFD mice. This effect was more robust in AD-T2D animals, supporting a more severe crosstalk between AD and overt diabetes and a major impact of the diabetic milieu on vascular impairment. While previous studies have supported anatomical alterations and changes in the vasculature of diabetic mice [31], to our knowledge, this is the first in vivo assessment of the actual functional changes that take place in the brain from prediabetic, T2D and AD models in real time. Vascular pulsation has been described as a mechanism of perivascular clearance of Aβ [49, 50] and as disease progresses; arteries show reduced vascular reactivity affecting perivascular drainage of fluids and solutes. CAA is associated with an increased relative pulsatility index in AD mice and patients [49]. Likewise, studies in old mice have shown reduced vessel wall pulsatility and capacity to clear Aβ [50] and similar observations have been reported after other insults [24, 51]. Vessel pulsation is determined by heartbeat and the role of the different components of the neurovascular unit, distinctively affecting arteries, arterioles and downstream microvessels. This activity is hampered by CAA, causing vascular tone deregulation, affecting vascular cells or impairing neurovascular coupling, ultimately interfering with perivascular clearance [52]. As CAA progresses, the neurovascular unit degenerates in amyloid-laden vessels [40, 53], affecting the integrity of the vessel wall and resulting in BBB leakage, vessel occlusion or rupture, leading to hemorrhage and decreased cerebral blood flow [2]. Also, under diabetic conditions, both the capillaries and larger vessels of the brain show a thickened basal lamina from collagen deposition, accumulated byproducts of lipid peroxidation, and deterioration of endothelial cells and pericytes [54].

Other studies have reported reduced evoked vascular reactivity in AD patients and transgenic mouse models of AD [55] that might also contribute to impaired perivascular clearance and subsequent Aβ deposition. In addition, lower blood flow velocity has been associated with dementia and cognitive decline in the Rotterdam study [56], and Aβ deposition is associated with impaired vascular reactivity responses in AD mice [57]. Likewise, our data are also in accordance with previous studies reporting that vasodilatation is compromised in aged AD mice [8]. Vascular functional alterations may result in CAA deposition, contributing to the loss of brain homeostasis and neuronal damage, but vascular damage may also contribute to CAA deposition by limiting Aβ clearance in a vicious cycle [58].

We further analyzed oxidative stress and MMP activation in vivo and in real time and we observed an overall increase of oxidative stress associated with amyloid deposition, along with metabolic alterations. Previous studies have largely documented increased oxidative stress in the brain and peripheral tissues in prediabetes and T2D. Chronic hyperglycemia and hyperinsulinemia, inherent to prediabetes and T2D, favor the formation of advanced glycation end products and the overproduction of reactive oxygen species, leading to extracellular matrix damage [59]. MMPs are required for the integrity of the extracellular matrix and have been associated with alterations in BBB permeability and neuropathological disorders [60]. We observed that MMP activation is significantly increased in association with CAA in AD mice with metabolic disorders. Since DQ^™^ Gelatin, used as a probe in our study, is preferentially degraded by MMP9, but it can also be degraded by MMP2 [61], we cannot unequivocally point towards one of them. Nevertheless, previous studies have shown that the increase of MMP2 and MMP9 activities is associated with the inflammatory process and oxidative stress in APP/PS1 mice [6, 9] and hyperglycemia alters the PKC-β pathway, causing an increase in MMP2 [62]. In addition, in patients with CAA a disbalance in MMP expression seems to be associated with CAA-related hemorrhages [45] and similar outcomes are observed in diabetic models [63].

We observed an overall increase in oxidative stress associated with amyloid plaques in AD-HFD and AD-T2D mice. Nevertheless, when CAA was analyzed, increased oxidative stress only reached statistical significance if overt diabetes was established. Whereas the amount of CAA is still limited in APP/PS1 by 7 months of age, the fragility of APP/PS1xdb/db mice precludes chronic in vivo studies at later stages. The fact that the presence of CAA is still scarce, limits the capacity of our approach to robustly discern between different type of vessels, althoughs the function and compromise of the vasculature and amyloid deposition is largely depending on the type of vessel under study; capillaries, penetrating arteries or leptomeningeal vessels.

On the other hand, we have previously observed the presence of microhemorrhages in the postmortem analysis of APP/PS1xdb/db mice [14, 19] and, indeed, microhemorrhages have been reported in close association with CAA [64]. Previous studies have shown an increased CAA severity in blood vessels involved in microinfarcts, whereas those involved in microhemorrhages had reduced CAA at the site of the bleeding [65]. It is then feasible that the combination of CAA with T2D, that affects vessel wall integrity and blood flow concomitantly, contributes to increased hemorrhage burden in this model [19]. In line with this idea, previous studies have shown that reactive oxygen species might be implicated in the formation of CAA and underlie CAA-related microhemorrhages in aged AD mice [8]. Thus, increased levels of reactive oxygen species lead to alterations in blood flow resistance and endothelial regulatory response is impaired by the disruption of endothelium-dependent nitric oxide signaling [66–68], resulting in increased microvessel permeability and impaired microvascular endothelial function [67, 69–71]. Hydrogen peroxide, superoxide derivates, hydroxyl radical or peroxynitrites increase endothelial calcium concentration in time- and dose-dependent manner causing dysregulation in calcium homeostasis, acting as proapoptotic factor in cerebral vascular cells [66, 70, 72]. In addition, oxidative stress has a key role in insulin resistance and progression of diabetes [73, 74] and diabetic mice show vascular alterations associated with increased oxidative stress, pericyte dysfunction and cerebrovascular integrity compromise [75]. Likewise, advanced glycation end products, resulting from hyperglycemia in T2D, alter the structure and function of BBB proteins [62] and diabetes-induced inflammation leads to astrocytic feet swelling in small vessels, separation of the plasma membrane and accumulation of hypertrophic microglia [54].

Our in vivo observations were also confirmed by *postmortem* assessment of oxidative stress and an overall increase of lipid peroxidation was observed in AD-T2D animals, when compared with AD alone. It should be noted that oxidative stress alters lipid-lipid interaction leading to impaired membrane permeability [76] and high levels of markers of oxidative stress-induced lipid peroxidation and cytotoxicity such as 4-hydroxynonenal, MDA or thiobarbituric acid reactive substances are increased in AD [77, 78] and diabetes [79, 80] supporting a synergistic effect when both diseases coexist. In addition, lipid peroxidation is accompanied by structural and functional alterations of the microvasculature [69], that may ultimately contribute to the observed pathology when AD and T2D coexist.

## Conclusions

Limitations of this study include the use of isoflurane anesthesia, which affects vessel function. Nevertheless, our data show that prediabetes accelerates amyloid pathology, suggesting that reversing the early stages of metabolic disease could slow down brain pathology. Functional alterations in the brain vasculature are significantly worsened in AD mice with T2D. Moreover, the presence of T2D has also a relevant impact on oxidative stress associated with amyloid deposition, both as amyloid plaques and CAA, providing further support to vascular pathology as underlying mechanisms linking T2D and AD.

## Data Availability

Data are available on reasonable request.

## Abbreviations

8-OHdG: 8-Hydroxy-2'deoxyguanosine
Aβ: Amyloid-β
AD: Alzheimer´s disease
BBB: Blood–brain barrier
HFD: High-fat diet
MDA: Malondialdehyde
MMP: Matrix metalloproteinases
RBC: Red blood cell
RD: Regular diet
T2D: Type 2 diabetes
